# A dose increased once-weekly bortezomib-based combination therapy for multiple myeloma

**DOI:** 10.18632/oncotarget.12162

**Published:** 2016-09-21

**Authors:** Daolin Wei, Yin Tong, Haitao Bai, Qi Cai, Yanrong Gao, Chun Wang

**Affiliations:** ^1^ Department of Hematology, Shanghai General Hospital, School of Medicine, Shanghai Jiao Tong University, Shanghai, 200080, China

**Keywords:** multiple myeloma, bortezomib, response rate, adverse event

## Abstract

**Background:**

The purpose of the current study was to evaluate the efficacy and safety of a dose increased weekly Bortezomib (Bor) based combination therapy in multiple myeloma (MM) patients.

**Results:**

The overall response rate (ORR) in the modified Bor group was 76.6%, composed of 40% complete response (CR), 3.3% very good partial response (VGPR) and 33.3% partial response (PR). The ORR was 82.3%, with 26.5% CR, 5.9% VGPR and 50% PR in control. A subgroup analysis showed both groups had equal efficacy in newly diagnosed MM patients (*P* = 1.000). The median progression free survival was 16 (11.7–20.3) months for the modified Bor group and 12 (10.5–13.5) months for the control (*P* = 0.503), and the median overall survival was 36 (9.4–62.6) vs 28 (21.6–34.4) months (*P* = 0.759). The incidences of AEs were similar except grade 1–4 peripheral neuropathy (PN) rate was 10% in modified regime group and 32.4% in control (*P* = 0.038).

**Materials and Methods:**

This was a monocentric, prospective, non-randomized, phase IV, non-inferiority trial. Thirty MM patients were treated with modified Bor-based combination therapy (Bor 1.6 mg/m^2^ on day 1, 8), with 34 MM patients on conventional Bor-based combination therapy (1.3 mg/m^2^ on day 1, 4, 8, 11) as control. The responses and adverse events (AEs) were compared.

**Conclusions:**

The increased-dose weekly Bor-based combination therapies were not inferior to conventional ones in terms of response and survival benefit, but showed lower rate of peripheral neuropathy (PN).

## INTRODUCTION

The application of novel drugs like Bortezomib (Bor) improved the clinical outcome and survival of multiple myeloma (MM) patients. Bor not only targets the myeloma cells, it also modulates the bone marrow environment through modulation of stromal cells and osteoblasts [1]. The application of Bor in MM started as monotherapy in patients with relapsed and refractory MM (RRMM), but a synergistic effect between Bor and corticosteroids has also been discovered [2–4]. The significant improvement of responses and overall survival for RRMM patients on Bor arm was confirmed in a randomized phase III trials [5, 6]. The combination of Bor with other agents was also examined, with synergistic effects confirmed [7–9]. Bor based combination therapy has been the frontline induction therapy in MM patients whether autologous stem cell transplantation (ASCT) candidate or not [10–12]. Bor has also been administered as monotherapy or in combination in other hematologic malignancies including Mantle cell lymphoma and Waldenström's Macroglobulinemia (WM) [13–15].

Although the prominent efficacy of Bor has been widely accepted, its major adverse events (AEs) were also noted, including thrombocytopenia, neutropenia, herpes zoster, fatigue, gastrointestinal toxicity and also peripheral neuropathy (PN), the most important and extensively studied Bor-related toxicity [3–5, 16]. Often these adverse events are so severe that lead to efficacious treatment discontinuation. Efforts have been made to balance its efficacy and toxicity, available strategies includes lower Bor single dose, weekly schedules and subcutaneous administration, but studies with conclusive evidence are lacking [17–19].

Under such circumstance, we explored a novel strategy of Bor administration in MM patients through increasing single dose and reduction in the frequency of administration, its efficacy and the concomitant influence on AEs were evaluated.

## RESULTS

### Patient characteristics

Thirty patients were enrolled into the modified Bor group and another thirty-four into the conventional Bor group as control. The characteristics of patients in both cohorts were compared and summarized in Table 1. The median age was similar between two groups, with 66 (37–86) for the modified group and 55 (42–76) for the control group. No significant differences were found in the age, gender, M-component type and disease staging between two groups. There were 24 newly diagnosed and 6 pre-treated MM patients in the modified Bor group including 2 relapsed and refractory multiple myeloma (RRMM) cases, and 30 newly diagnosed and 4 pre-treated cases (none RRMM) in the control group. There was no distribution bias observed (Table 1).

**Table 1:** Comparison of multiple myeloma patients characteristics

Character	Modified Bor group, *n* = 30	Conventional Bor group, *n*= 34	*P*
Gender, *n*(%)			1.000
M	16 (53.7)	19 (55.9)	
F	14 (46.7)	15 (44.1)	
M-component, (*n*/%)			0.172
IgG	12 (40.0)	19 (55.9)	
IgA	8 (26.7)	7 (20.6)	
IgD	3 (10.0)	6 (17.6)	
Light chain	7 (23.3)	2 (5.9)	
D-S stage, (*n*/%)			0.616
II	1 (3.3)	3 (8.8)	
III	29 (96.7)	31 (91.2)	0.369
ISS stage, (*n*/%)			
I	4 (13.3)	6 (17.6)	
II	10 (33.3)	16 (47.1)	
III	16 (53.3)	12 (35.3)	
Treatment history, (*n*/%)			0.495
Untreated	24	30	
Pre-treated	6	4	

### Efficacy assessment

The complete response (CR) rate was 40% in the modified Bor group vs 26.5% in the control group (*P* = 0.294) and the very good partial response (VGPR) rate was 3.3% vs 5.9% (*P* = 1.000). The partial response (PR) rate was 33.3% vs 50.0% (*P* = 0.211) and the overall response rate (ORR) was 76.6% vs 82.3% (*P* = 0.757). There was no statistical difference in the treatment efficacy between the modified Bor group and the control (Table 2). The median time to response (TTR) was 4 (4–12) weeks in the modified Bor group and 4 (3–16) weeks in the control, respectively. A subgroup analysis based on newly diagnosed and pre-treated patients showed no significant efficacy difference (*P* = 1.000) between two regimen group (Table 3). One RRMM case in the modified Bor regimen group died before the end of the first course and the response was categorized as progression disease (PD).

**Table 2:** Efficacy of Bor-based regimens for MM patients

Responses	Modified Bor group, *n* = 30	Conventional Bor group, *n*= 34	*P*
ORR, *n*(%)	23 (76.6)	28 (82.3)	0.757
CR, (*n*/%)	12 (40)	9 (26.5)	0.294
VGPR, (*n*/%)	1 (3.3)	2 (5.9)	1.000
PR, (*n*/%)	10 (33.3)	17 (50)	0.211
SD, (*n*/%)	6 (20)	5 (14.7)	0.742
PD, (*n*/%)	1 (3.3)	1 (2.9)	1.000

**Table 3:** Efficacy results of Bor-based therapy in pre-treated and untreated MM patients

Treatment history	Responsive, *n* (%)	Unresponsive, *n* (%)	*P*
Untreated			1.000
Modified Bor group, *n* = 24	20 (83.3)	4 (16.7)	
Conventional Bor group, *n*= 30	26 (86.7)	4 (13.3)	
Pre-treated			1.000
Modified Bor group, *n* = 6	3 (50)	3 (50)	
Conventional Bor group, *n*= 4	2 (50)	2 (50)	

### Survival analysis

**Figure 1:**
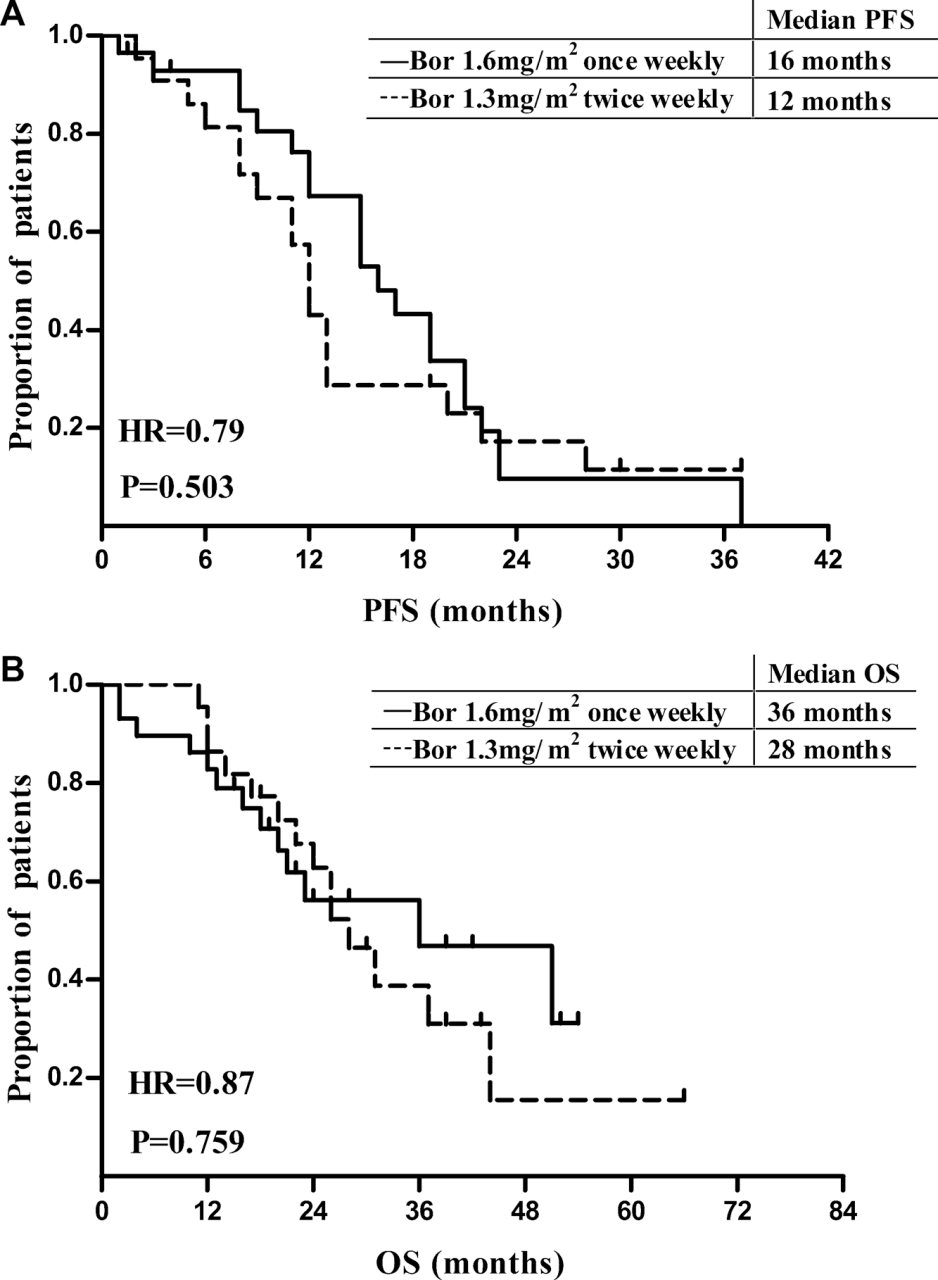
Progression-free survival and overall survival of MM patients treated with Bor based regimens

It is obvious that the high dose therapy and ASCT are associated with additional survival benefit. In order to avoid its interference on survival analysis, thirteen patients (1 in modified group, 12 in control group) after ASCT were excluded from the survival analysis. By the end of the follow-up, the median time of follow-up was 21 (1–54) months for the modified Bor group, and 26 (11–66) months for the conventional Bor group. Thirteen cases died in the modified Bor group, with 14 cases died in the control group. The difference in the accumulated death rates (44.8% vs 63.6%) was of no statistical significance (*P* = 0.259). Patients achieved similar survival benefit from both Bor-based regimens. The median PFS was 16 (11.7–20.3) months in the modified group vs 12 (10.5–13.5) months in the control group (*P* = 0.503) (Figure 1A). The median OS was 36 (9.4–62.6) months vs 28 (21.6–34.4) months (*P* = 0.759), the one-year cumulative survival rates were 95% and 86% for modified and conventional Bor group (Figure 1B).

### Treatment-related toxicity

The median courses received were 4 (1–10) for the modified Bor group and 4 (1–7) for the control, the average cycles received were 3.9 vs 3.6, the median cumulative Bor dosage were 12.8 mg/m^2^ in the modified Bor group vs 20.8 mg/m^2^ in the control, respectively. Treatment-related AEs with both Bor regimen was summarized (Table 4). The major AEs included but not restricted to peripheral neuropathy (PN), herpes zoster, dyspnea, etc. The general occurrence rates were similar in two groups except the overall PN was less in modified regime group (10% vs 32.4%, *P* = 0.038). Grade 3–4 PN rate was also less in the modified Bor group, but did not reach statistically significance (3.3% vs 14.7%, *P* = 0.202). Five cases in conventional Bor group stopped Bor treatment due to grade 3 or higher neuropathy, and 3 re-initiated Bor therapy after management, 2 failed to show any improvement and caused discontinuation of Bor therapy. While only 1 patient in modified Bor group stopped treatment due to grade 3 neuropathy but re-initiated Bor therapy after neuropathy symptom resolved by management. There were also more herpes zoster and gastrointestinal symptoms occurred in the conventional regimen group, but reached no statistically significance. One case in each group experienced severe dyspnea. No pulmonary abnormal signs were found in Computer Tomography examination for the patient with modified Bor regimen, the symptom resolved spontaneously. On the contrary, diffuse pulmonary abnormalities were found in Computer Tomography examination for a patient from conventional Bor regimen group, the symptom resolved after corticoid administration.

**Table 4:** AEs observed in patients treated with Bor

	Modified Bor group *n* = 30		Conventional Bor group *n* = 34		*P*
	Grade 1–4	Grade 3–4	Grade 1–4	Grade 3–4	
Neuropathy, (*n*/%)	3 (10)	1 (3.3)	11 (32.4)	5 (14.7)	0.038
Thrombocytopenia, (*n*/%)	2 (6.6)	1 (3.3)	2 (5.9)	1 (2.9)	1.000
Neutropenia, (*n*/%)	2 (6.6)	1 (3.3)	3 (8.8)	1 (2.9)	1.000
Herpes zoster, (*n*/%)	3 (10)	2 (6.6)	5 (14.7)	2 (5.8)	0.713
Dyspnea, (*n*/%)	4 (13.3)	1 (3.3)	4 (11.8)	1 (2.9)	1.000
Gastrointestinal, (*n*/%)	2 (6.6)	1 (3.3)	5 (14.7)	2 (5.9)	0.433

## DISCUSSION

Several trials of modified Bor-based combination therapy were reported. Palumbo et al. carried out a phase 3 trial in patients with newly diagnosed MM who were not eligible for high-dose therapy followed by ASCT. But the protocol was amended and patients received once-weekly intravenous bortezomib instead of the initial twice-weekly bortezomib infusions for safety concerns. The CR rate was 30% in the once-weekly group and 35% in the twice-weekly group (*P* = 0.27). Similar long-term outcomes were also observed for 3-year PFS (50% vs 47%, *P* = 1.00) and OS (88% vs 89%, *P* = 0.54) [18, 20]. Mateos et al. investigated a bortezomib-based regimen in elderly patients with untreated MM [17]. Patients were randomly assigned to receive VMP or VTP as induction therapy, consisting of one cycle of bortezomib twice per week for 6 weeks, plus either melphalan or daily thalidomide, and prednisone, followed by five cycles of bortezomib once per week for 5 weeks plus the same doses of melphalan plus prednisone and thalidomide plus prednisone. 81% patients in the VTP group and 80% in the VMP group achieved PR or better responses (*P* = 0.90), with similar CR rates (28% vs 20%, *P* = 0.20) after the six cycles of induction therapy. The response rates were close to previous report. These two reports showed the reduction of Bor administration frequency (twice-weekly to once-weekly) in combination therapy did not compromised its efficacy.

There were some reports that examined the effect of increased-dose Bor. A phase 2 trial examined a 3-drug combination of bortezomib, cyclophosphamide, and dexamethasone (CyBorD) in newly diagnosed MM patients [21]. Cohort 1 received 1.3 mg/m^2^ of bortezomib intravenously on days 1, 4, 8 and 11, and cohort 2 received 1.5 mg/m^2^ of bortezomib intravenously on days 1, 8, 15, 22 instead. The results showed the overall response rates were similar in cohort 1 & 2 (88% vs 93%). Ghobrial et al. conducted a phase 1/2 study that assessed the response and safety of the combination of temsirolimus and bortezomib in RRMM patients [22]. The maximum tolerated dose was determined to be 1.6 mg/m^2^ bortezomib on days 1, 8, 15, and 22, in combination with 25 mg temsirolimus on days 1, 8, 15, 22, and 29, for a cycle of 35 days. The proportion of patients with a partial response or better was 33%.

In this study, we examined the response of modified weekly, increased-dose Bor based combination therapy in MM patients. The majority of both current cohort were newly diagnosed MM patients, the rest were pre-treated MM patients including some RRMM. The overall response (PR or higher) rates in the modified Bor group were comparable to those in the conventional Bor group (76.6% vs 82.3%, *P* = 0.757), whether as primary therapy for newly diagnosed patients (83.3% vs 86.7%, *P* = 1.000) or as salvage/second line therapy for pre-treated patients (50% vs 50%, *P* = 1.000). The median TTR and rates of CR, VGPR, PR were also quite similar. After exclusion of patients who underwent ASCT, survival analysis showed that the median PFS (16 vs 12 months, *P* = 0.503) and OS (36 vs 28 months, *P* = 0.759) were close between two groups. The similar response rate and survival benefit suggested the efficacy of modified Bor regimens was equivalent or inferior to that of conventional Bor regimens. Considering the median cumulative Bor dosage (12.8 mg/m^2^ vs 20.8 mg/m^2^), the modified weekly, increased-dose Bor-based regimen was actually more cost-effective. However, due to the limited sample size, caution should be taken when interpreting the results especially concerning the pre-treated subgroup. A large scale, randomized clinical trial is needed to confirm our finding.

As for the AEs, fewer peripheral neuropathy occurred in the modified Bor group (10% vs 32.4%, *P* = 0.038), the severity was also reduced when compared with the conventional Bor group even without statistically significance. The reduction of PN incidence here was similar to reports (5–8%) of APEX and VISTA trial, both trials showed the weekly Bor schedules resulted in a reduction of AEs compared with the conventional twice weekly schedule [5, 10]. Also in another report, non-hematologic grade 3 to 4 AEs were more frequent in patients on twice-weekly bortezomib than those received once-weekly bortezomib (51% vs 36%, *P* < 0.003), which was mainly related to reduction of severe sensory peripheral neuropathy (16% vs 3%, *P* < 0.001) [18]. The occurrences of hematologic toxicity, herpes zoster, and gastrointestinal disorders were similar in both groups. The hematologic toxicities were generally mild, never exceeding grade 3, and reversible after introduction of stimulating agents like G-CSF and IL-11, without obvious infection or bleeding symptoms developed. The gastrointestinal disorders included diarrhea, abdomen pain, liver malfunction, resolved in both groups with certain medication. There was also more herpes zoster occurred in the conventional regimen group (4 vs 1), but it did not reach statistically significance. The safety assessment suggested this weekly, dose intensified Bor regimen reduced peripheral neural toxicity and could be well tolerated by patients with MM.

Bortezomib administration modification has also been attempted in follicular lymphoma and WM [14, 15]. In both trials, Bor was administered intravenously at a dose of 1.6 mg/m^2^ once weekly combined with agents like rituximab and bendamustine in relapsed or refractory follicular lymphoma or newly diagnosed WM. The safety analysis showed the Bor related neurotoxicity incidences were reduced.

In current study, the modified bortezomib-based (1.6 mg/m^2^, intravenously on day 1, 8, regimens were not inferior to conventional ones (1.3 mg/m^2^, intravenously on day 1, 4, 8, 11) in terms of responses, survival benefit and AEs incidence.

## MATERIALS AND METHODS

### Patients

The first cohort composed of 30 symptomatic MM patients with measurable para-protein treated with modified schedules of Bor based regimens at the Shanghai General Hospital from July 2010 to February 2014. Another cohort included 34 MM patients who were treated with standard schedules of Bor concurrently. Before enrolling in this trial, all patients were evaluated with a complete history and physical examination (including neurologic examination), complete blood counts and differential, chemistry profile, serum b2–microglobulin level, serum or urine electrophoresis, quantitative immunoglobulins, and metastatic bone survey. All patients were diagnosed according to the International Myeloma Working Group (IMWG 2003) criteria, Durie-Salmon (D-S) and International Staging System (ISS) [23]. The study was approved by the institutional review board and all patients provided informed consent (The study is registered at http://www.clinicaltrials.gov as NCT02559154).

### Study design and treatment

This cohort study is a monocentric, prospective, non-randomized, phase IV, non-inferiority trial. The primary end point was objective response rate (CR, very good PR, and PR) within 4 cycles of Bortezomib based regimen treatment. The second end point was the incidence of adverse events. Patients in the modified Bor group received regimens with Bor (1.6 mg/m^2^) as an intravenous bolus once weekly on day 1, 8 in a 3 weeks cycle, in combination with dexamethasone, with or without doxorubicin/cyclophosphamide/mitoxantrone/thalidomide, while patients in the conventional Bor group received similar combination therapy except with standard Bor (1.3 mg/m^2^) administration twice weekly on day 1, 4, 8, 11. Patients were treated until best response reached or Bor discontinued due to severe AEs or patient death. Therapy after best response included thalidomide plus dexamethasone, cyclophosphamide plus thalidomide and dexamethasone, autologous stem cell transplantation (ASCT). Acyclovir was administered for herpes prophylaxis, but prophylactic anti-coagulation agent was not routinely used unless the patient had prior thrombosis.

### Response and safety assessment

Responses were evaluated after each treatment cycle according to the IMWG Uniform Response Criteria [24], partial response and higher responses were calculated as overall response. The overall response rate (ORR), progression-free survival (PFS) and overall survival (OS) were compared. PFS was measured from the date of the first dose of Bor administration until the date when myeloma progression was documented. OS was defined as the time from the date of first Bor administered until the date of death from any course. All patients were followed until January-31st 2015. Time to treatment response (TTR) was calculated from the first Bor injection until a partial or higher response was obtained. All patients received at least 1 dose of Bor were evaluated for toxicity using the Nation Cancer Institute Toxicity Criteria (Version 3.0) [25].

### Statistical analysis

Two cohorts were compared for the response category and the incidence of adverse events using the chi-square test. The TTR, PFS, and OS analysis were carried out with the Kaplan-Meier method, and 95% confidence intervals (CI) were calculated. A log-rank test was used for comparisons of TTR, PFS, and OS between two groups for statistically significance. Results were considered significant if the *P value* was less than 0.05. Statistical analyses were performed using the SPSS System (Version 17.0).
